# The COVID-19 pandemic has not influenced survival outcomes of head and neck cancer squamous cell carcinomas in the West of Scotland: a retrospective cohort study

**DOI:** 10.1038/s44276-026-00203-3

**Published:** 2026-03-04

**Authors:** Craig D. L. Smith, Alex D. McMahon, Gareth J. Inman, David I. Conway, Catriona M. Douglas, Claire Paterson

**Affiliations:** 1 School of Medicine, Dentistry, and Nursing, University of Glasgow, Glasgow, UK; 2 Glasgow Head and Neck Cancer (GLAHNC) Research Group, Glasgow, UK; 3 Cancer Research UK Scotland Institute, Glasgow, UK; 4 Department of Otolaryngology/Head and Neck Surgery—Glasgow Royal Infirmary and Queen Elizabeth University Hospital, Glasgow, UK; 5 Beatson West of Scotland Cancer Centre, Gartnavel General Hospital, Glasgow, UK

## Abstract

**Background:**

The COVID-19 pandemic posed significant disruptions to healthcare worldwide. This study aimed to assess if the COVID-19 pandemic affected short-term survival outcomes of patients with head and neck cancer squamous cell carcinoma (HNSCC) in the west of Scotland.

**Methods:**

A retrospective cohort analysis was conducted using regional centre data. Patients diagnosed with HNSCC during pre-pandemic (June-October 2019), intra-pandemic (June-October 2020) and post(peak)-pandemic (June-October 2022) were compared. Demographic, behavioural, clinical, and survival data were analysed using Kaplan-Meier curves, Cox-regression, and logistic-regression for stage at diagnosis.

**Results:**

A total of 707 patients were included. While the 2020 cohort exhibited an increase in advanced-stage disease (67.5% *p* = 0.01), no statistically significant difference in overall survival was observed across the study period (*p* = 0.22). Cox-regression showed no associations between year of diagnosis and mortality, even after multivariable adjustment for confounders. Survival was strongly associated with age, performance status, stage, and HPV status among oropharynx cancers. Socioeconomic inequalities persisted, with the poorest survival among those from the most socioeconomically deprived areas (*p* = 0.02).

**Conclusions:**

While an increase in advanced-stage HNSCC diagnoses was observed in the West of Scotland during 2020, short-term survival outcomes remained comparable to pre- and post-pandemic.

## Introduction

Head and neck cancer (HNSCC), commonly defined as cancers of the oral cavity, pharynx and larynx, is associated with poor survival at advanced stages[1]. Research has emphasised that timely diagnosis and treatment are crucial for achieving optimal survival and quality of life outcomes[2]. The COVID-19 pandemic, caused by the SARS-CoV-2 virus, inflicted great stress and disruptions on healthcare systems worldwide, including the UK[3, 4]. In the acute phases of the pandemic in 2020 and 2021, public health guidance advised primary and secondary care medical and dental care services to postpone all non-urgent face-to-face consultations and elective procedures. The population was advised not to access healthcare unless for urgent or emergency care.

Delays and declines in the number of referrals to head and neck units were subsequently observed across the UK[5–7]. These were accompanied by concerns that the reduced quantity of referrals would result in an increased burden of advanced-stage disease, with detrimental prognostic implications[8].

A subsequent rapid review and meta-analysis of the wider international literature suggested a shift towards advanced-stage diagnoses associated with the COVID-19 pandemic[9]. Currently, there is little research assessing the longitudinal effects of the COVID-19 pandemic on HNSCC survival outcomes. Using retrospective data collected from routinely diagnosed and treated cohorts, this analysis aimed to identify if HNSCC survival in the west of Scotland was adversely affected by the COVID-19 pandemic.

## Methods

Local clinical NHS Caldicott Guardian and University of Glasgow ethical (Project number 200240340) approvals were obtained. HNSCC cases were identified from the regional multidisciplinary team (MDT) database, which records all new HNSCC cases in the west of Scotland region. This west of Scotland cancer network region serves a population of 2.5 million people (approximately 46% of the Scottish population)[10]. Demographic, behavioural and clinical data were collected from electronic patient records. This retrospective cohort analysis was conducted as per the Strengthening the Reporting of Observational Studies in Epidemiology (STROBE) framework[11].

### Inclusion and exclusion criteria

Participants were defined as patients with histologically confirmed squamous cell carcinomas of the oral cavity (C00.3 - C00.9, C02–C05), oropharynx (C01, C02.4, C09, C10, C14), larynx (C32), nasopharynx (C11), Hypopharynx (C12, C13) and other overlapping sites (C14.8)[12, 13]. Participants were excluded if they had diagnoses of recurrent cancers, tumours of the thyroid or salivary glands, or haematological cancers of the head and neck.

### Data collection

Patients were grouped into three cohorts according to their time of diagnosis. These comprised a “pre-pandemic” cohort (1st June to 31st October 2019), an “intra-pandemic” cohort (1st June to 31st October 2020) and a “post-pandemic” cohort (1st June to 31st October 2022). This latter period constituted a time where most public health measures (including mask-wearing and travel restrictions) had been relaxed in Scotland following the rollout of the national vaccination programme and declines in COVID-19 incidence, although the World Health Organisation did not declare an official end to the global pandemic until the 5th of May 2023[14, 15]. Follow-up was conducted from the date of first MDT to the 8th of March 2025

Demographic data included patients’ age at diagnosis, sex, and home area-based socioeconomic deprivation level. The Scottish Index of Multiple Deprivation (SIMD) is an area-based socioeconomic deprivation score derived from postcode data zones. The score considers seven key domains, including access to services, crime, education, employment, healthcare, housing and income[16]. This was reported in fifths, with SIMD-1 corresponding to the 20% most socioeconomically deprived of the population and SIMD-5 corresponding to the 20% least socioeconomically deprived.

Clinical information included cancer subsite, HPV status for oropharynx cancers (all suspected oropharynx cancers undergo p16 testing as a surrogate marker for tumour HPV status), AJCC stage (I-IV), Disease (early; I and II/advanced; III and IV), ECOG (Eastern Cooperative Oncology Group) Performance Status (PS 0-4, 0 = fully active; 1 = Restricted in physically strenuous activity but ambulatory; 2 = Ambulatory and capable of all selfcare but unable to carry out any work activities; 3 = Capable of only limited selfcare; confined to bed or chair more than 50% of waking hours; 4 = Completely disabled; cannot perform any selfcare), emergency tracheostomy (Yes/No), treatment intent (curative/palliative) and treatment modality delivered (Best Supportive Care (BSC)/Palliative Systemic Anti-Cancer Therapy (SACT)/Radiotherapy (RT) alone/Chemoradiotherapy (CRT) / Surgery alone/Surgery + RT/Surgery + CRT)[17].

Behavioural data included smoking history status (ever/never) and alcohol excess consumption status (ever/never)—defined as greater than 14 units of alcohol per week[18].

### Statistical analysis

Descriptive statistics were calculated and reported for the pre-pandemic, intra-pandemic, and post-pandemic cohorts. Following normality testing, one-way ANOVA tests were calculated for continuous variables. Pearson’s chi-squared tests of association or Fisher’s exact tests were used to assess for associations among categorical demographic, behavioural, and clinical variables (including treatment delivery).

Survival was defined as the time between diagnosis and death of any cause. Kaplan-Meier analysis was used to compare the survival of the cohorts over the study period. Unadjusted and adjusted Cox regression analysis was also conducted, using survival time as the timescale to calculate Hazard Ratios (HR) with 95% Confidence Intervals (95% CI).

Logistic regression analysis was also conducted for stage of presentation (early/advanced), with Odds Ratios (OR) and 95% Confidence Intervals (95% CI) calculated. Test results were considered statistically significant where *p* < 0.05. All these analyses were conducted on R-Studio v.4.4.3.

## Results

A total of 707 patients were included in this study: 239 from the 2019 “pre-pandemic” cohort, 237 from the 2020 “intra-pandemic” cohort and 231 from the 2022 “post-pandemic” cohort (Table 1). There was no statistically significant difference in the number of cases in each 6-month time period among the 2019, 2020 and 2022 cohorts. (*p* = 0.93).

**Table 1:** Descriptive Statistics of the 2019, 2020 and 2022 Cohorts.

	2019 (*n* = 239)	2020 (*n* = 237)	2022 (*n* = 231)	*p*
Total	239	237	231	0.93^a^
Follow up, Person-Years (Mean ± SD)	3.1 ( ± 2.4)	2.6 ( ± 1.9)	1.8 ( ± 1.0)	
Events (as of 8.3.25)				**<0.01**^**a**^
Alive / Censored	109 (45.6%)	100 (42.2%)	132 (57.1%)	
Deceased	130 (54.4%)	137 (57.8%)	99 (42.9%)	
Site				**<0.01**^**a**^
Oral Cavity	54 (22.6%)	84 (35.4%)	56 (24.2%)	
Oropharynx	77 (32.2%)	73 (30.8%)	81 (35.1%)	
Larynx	72 (30.1%)	44 (18.6%)	60 (26.0%)	
Nasopharynx	10 (4.2%)	6 (2.5%)	4 (1.7%)	
Hypopharynx	22 (9.2%)	16 (6.8%)	20 (8.7%)	
Cancer of Unknown Primary (CUP)	4 (1.7%)	14 (5.9%)	10 (4.3%)	
Oropharynx ca. HPV Status				0.43^b^
P16 Positive	49 (63.6%)	42 (57.5%)	56 (69.1%)	
P16 Negative	24 (31.2%)	28 (38.4%)	24 (29.6%)	
N.A.	4 (5.2%)	3 (4.1%)	1 (1.2%)	
Age at MDT, Years (mean ± SD)	65.2 ( ± 11.9)	64.7 ( ± 11.5)	65.4 ( ± 11.6)	0.77^c^
Sex				0.14^a^
Female	62 (25.9%)	79 (33.3%)	61 (26.4%)	
Male	177 (74.1%)	158 (66.7%)	170 (73.6%)	
SIMD Quintile				**0.01**^**a**^
1 (20% Most Deprived)	110 (46.0%)	78 (32.9%)	88 (38.1%)	
2	52 (21.8%)	63 (26.6%)	37 (16.0%)	
3	32 (13.4%)	38 (16.0%)	47 (20.3%)	
4	21 (8.8%)	37 (15.6%)	33 (14.3%)	
5 (20% Least Deprived)	24 (10.0%)	21 (8.9%)	26 (11.3%)	
Disease				**0.01**^**b**^
Early (Stage I and II)	103 (43.1%)	76 (32.1%)	101 (43.7%)	
Advanced (Stage III and IV)	136 (56.9%)	160 (67.5%)	127 (55.0%)	
Missing	0	1 (0.4%)	3 (1.3%)	
AJCC Stage				0.06^b^
I	68 (28.5%)	48 (20.3%)	58 (25.1%)	
II	35 (14.6%)	28 (11.8%)	43 (18.6%)	
III	48 (20.1%)	49 (20.7%)	42 (18.2%)	
IV (IV, IVa, IVb & IVc)	88 (36.8%)	111 (46.8%)	85 (36.8%)	
Missing	0	1 (0.4%)	3 (1.3%)	
Smoking history				0.60^b^
Never	52 (21.8%)	60 (25.3%)	57 (24.7%)	
Ever	187 (78.2%)	177 (74.7%)	173 (74.9%)	
Missing	0	0	1 (0.4%)	
History alcohol excess				0.98^b^
Never	158 (66.1%)	157 (66.2%)	152 (66.1%)	
Ever	81 (33.9%)	80 (33.8%)	78 (33.9%)	
Missing	0	0	1 (0.4%)	
ECOG PS				0.32^b^
0	103 (43.1%)	107 (45.1%)	122 (52.8%)	
1	74 (31.0%)	78 (32.9%)	60 (26.0%)	
2	38 (15.9%)	30 (12.7%)	33 (14.3%)	
3	23 (9.6%)	18 (7.6%)	14 (6.1%)	
4	1 (0.4%)	4 (1.7%)	2 (0.9%)	
Emergency Tracheostomy				0.37^a^
No	234 (97.9%)	230 (97.0%)	221 (95.7%)	
Yes	5 (2.1%)	7 (3.0%)	10 (4.3%)	
Treatment Intent				0.21^b^
Curative	155 (65.0%)	159 (66.7%)	163 (70.6%)	
Palliative	84 (35.0%)	78 (33.3%)	66 (28.6%)	
N.A.	0	0	2 (0.9%)	
Treatment Delivered				^d^0.63^a^
BSC	75 (31.4%)	73 (30.8%)	56 (24.2%)	
Palliative SACT	17 (7.1%)	10 (4.2%)	14 (6.1%)	
RT alone	34 (14.2%)	36 (15.2%)	38 (16.5%)	
CRT	39 (16.3%)	35 (14.8%)	38 (16.5%)	
Surgery alone	50 (20.9%)	44 (18.6%)	53 (22.9%)	
Surgery + RT	17 (7.1%)	27 (11.4%)	21 (9.1%)	
Surgery + CRT	7 (2.9%)	12 (5.1%)	11 (4.8%)	

Significance highlighted in **bold**.

*N.A.* Not applicable/assessed, *SIMD Quintile* Scottish index of Multiple Deprivation Quintile, *ECOG PS* Performance Status (0 = fully active; 1 = Restricted in physically strenuous activity but ambulatory; 2 = Ambulatory and capable of all selfcare but unable to carry out any work activities; 3 = Capable of only limited selfcare; confined to bed or chair more than 50% of waking hours; 4 = Completely disabled; cannot perform any selfcare), *BSC* Best Supportive Care, *CRT* Chemo-radiotherapy, *RT* Radiotherapy, *SACT* Systemic Anti-cancer Therapy.

^a^Pearson Chi-squared.

^b^Fishers Fisher’s exact (simulated).

^c^One-way ANOVA.

^d^When comparing surgical (surgery alone, surgery + RT or CRT) and oncology-centred (RT alone & CRT) treatments, *p* = 0.82 Pearson Chi-squared.

### Sociodemographic data

Patients among cohorts were of a similar mean age at the time of diagnosis (65.2 (2019), 64.7 (2020) and 65.4 (2022) years old). Most patients were male (71.4%, *n* = 505) across all three cohorts (*p* = 0.14). A strong socioeconomic gradient was observed, with 39% of patients living in SIMD-1 (20% most socioeconomically deprived) areas. This remained true across the study duration (46.0% (2019), 32.9% (2020), and 38.1% (2022)). A reduction in the proportion of patients from lower SES backgrounds (SIMD-1, 20% most deprived) was observed in 2020 (*p* = 0.01).

### Behavioural data

Most patients (*n* = 537, 76.0%) reported a current or former smoking status, which was uniform across the cohorts (*p* = 0.60). Similarly, many HNSCC patients (*n* = 239, 33.8%) reported a current or previous history of alcohol excess across the cohorts (*p* = 0.98).

### Clinical data

Advanced-stage disease was observed among 59.8% of patients (Table 1). When evaluating case distributions by AJCC stage, a narrowly non-significant difference was observed among the cohorts (*p* = 0.06). However, a significant difference in the distribution of cancer stages was detected upon grouping of stages into early (I and II) and advanced-stage disease (III and IV) (*p* = 0.01). When grouped into early- and advanced-stage disease, there was significant variation in the distribution across the study cohorts (*p* = 0.01). Specifically, the pandemic cohort had a higher proportion of advanced-stage disease (67.5%) compared with the 2019 (56.9%) and 2022 cohorts (59.0%).

Comparable functional status metrics (ECOG PS) were reported across the cohorts (p = 0.32), with most of the patients (*n* = 332, 47.0%) reported as Performance Status 0 (fully active) or 1 (restricted in physically strenuous activity but ambulatory and capable of light work, *n* = 212, 30.0%).

Most of the patients presented to the MDT across each cohort year with OCC (*n* = 194, 27.4%), OPC (*n* = 231, 32.7%) and larynx cancers (*n* = 176, 24.9%), with cancers of the hypopharynx, nasopharynx and unknown primaries constituting smaller numbers. While the proportions of cases were similar in 2019 and 2022, increases in the proportion of OCC cases and a concurrent reduction of larynx cancer cases were found among the 2020 cohort. Increases in the number of cancers of unknown primaries also occurred among the 2020 and 2022 cohorts. This was reflected in significance testing, where changes in subsite distributions were detected (*p* = 0.01). Most OPC cases (*n* = 223, 96.5%) had undergone p16 testing as a marker for HPV. The numbers of patients with HPV-positive OPC was comparable across the cohorts (*p* = 0.43), with 63.6% (*n* = 147) of OPC cases presenting with HPV positive tumours. Small numbers of patients required emergency tracheostomies across the cohorts (*n* = 22, 3.1%, *p* = 0.37), although the proportion of those with a need for an emergency tracheostomy in 2022 was double that of 2019.

Treatment decisions (curative versus palliative) at the multidisciplinary team level were consistent across the study duration (*p* = 0.21), with most patients in the cohorts selected for treatment approaches with curative intent (*n* = 477, 67.4%). The same uniformity was observed among treatment modalities delivered across the cohorts (*p* = 0.63), with no significant changes in the proportions of patients that received palliative, surgical, oncological or combination treatments. When comparing surgical vs oncology centred treatments, a similar non-significant finding was observed (*p* = 0.82)

### Overall survival

**Fig. 1:**
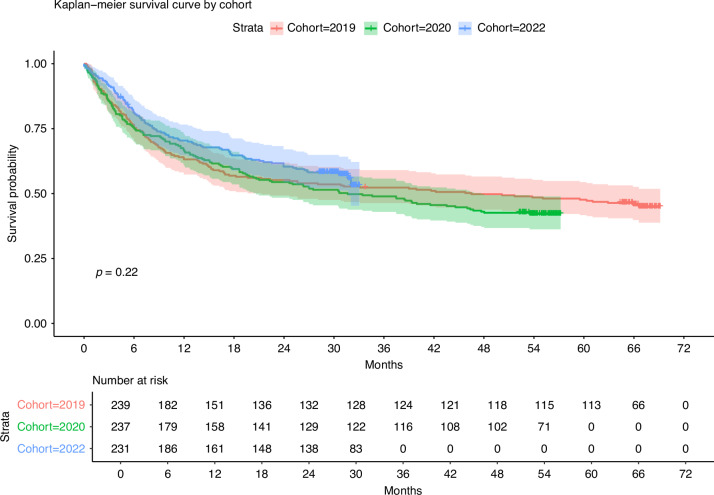
Kaplan-Meier Survival Curve (with Confidence Intervals) for patients diagnosed with Head and Neck Cancer in the west of Scotland during 2019 (*n* = 239, 130 events), 2020 (*n* = 237, 137 events) and 2022 (*n* = 231, 99 events)

Figure 1 presents the overall survival of the three cohorts throughout the study duration. During follow-up from the date of MDT to the 8th of March 2025, 130 (54.4%), 137 (57.8%) and 99 (42.9%) deaths were recorded among the 2019, 2020 and 2022 cohorts, respectively. Two-year overall survival was 55.2% (95% CI = 49.3–61.9) for 2019, 54.4% (95% CI = 48.4–61.2) for 2020 and 60.4% for 2022 (95% CI = 54.3–67.0). Kaplan-Meier analysis with log-rank testing revealed no statistically significant difference in overall survival (*p* = 0.22).

**Fig. 2:**
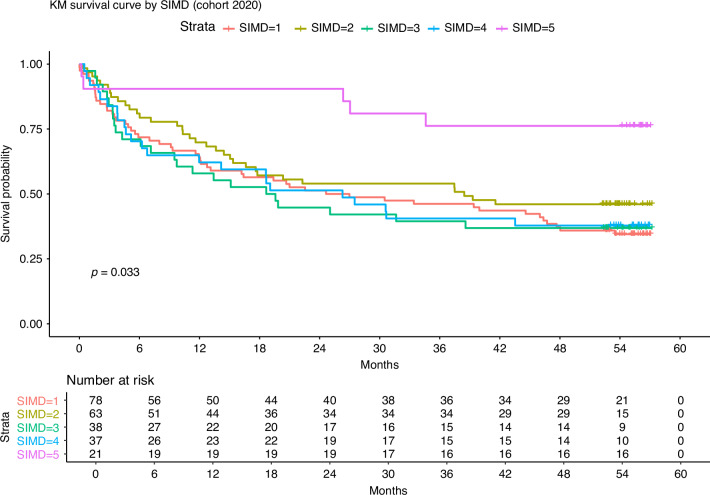
Kaplan-Meier Survival Curve stratified by SIMD (Scottish Index of Multiple Deprivation) for patients diagnosed with Head and Neck Cancer in the west of Scotland among the 2020 cohort. SIMD 1 = 20% Most deprived, SIMD 5 = 20% Least deprived.

Survival was socioeconomically patterned; two-year overall survival was 52.8% (95% CI = 47.2–59.0) for individuals in SIMD 1 (20% most deprived), 52.6% (95% CI = 45.3–61.2) for SIMD 2, 58.7% (95% CI = 50.4–68.4) for SIMD 3, 59.3% (95% CI = 50.1–70.3) for SIMD 4, and 73.2% (95% CI = 63.6–84.3) for SIMD 5 (20% least deprived). Kaplan-Meier analysis with log-rank testing revealed a statistically significant difference in overall survival across SIMD quintiles (Supplementary Fig. 1, *p* = 0.02). When analysing SIMD fifths by cohort year, no survival difference was detected among patients from the 2019 cohort (Supplementary Fig. 2, *p* = 0.58). A wide and statistically significant difference in overall survival was observed among the 2020 cohort when assessing SIMD fifths, with wide survival inequalites among those living in the most (SIMD-1) and least (SIMD-5) socioeconomically deprived areas (Fig. 2, *p* = 0.033). This widening of survival inequalities persisted into 2022, with large variations in survival observed by SIMD fifth (Supplementary Fig. 3, *p* = 0.017).

**Fig. 3:**
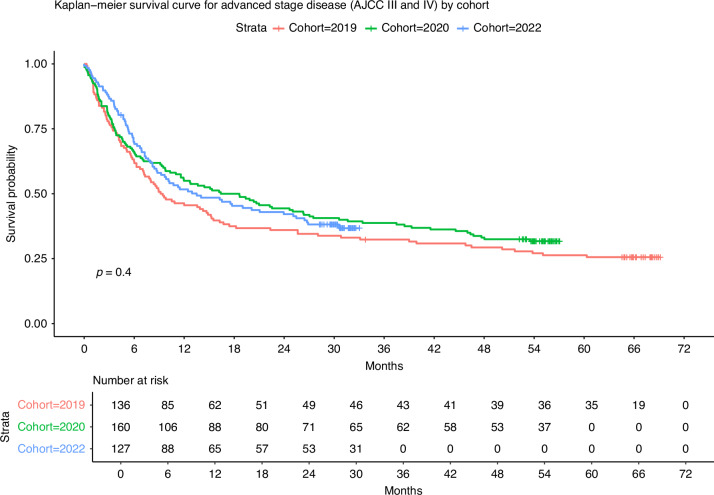
Kaplan-Meier survival curve for patients diagnosed with advanced stage (AJCC Stage III and IV) Head and Neck Cancer in the west of Scotland among the 2019, 2020 and 2022 cohorts.

Diagnosis at advanced-stage disease, relative to early-stage, was associated with deleterious survival (Supplementary Fig. 4, *p* < 0.0001). No differences in overall survival were observed for advanced-stage (III and IV) disease among the cohorts (Fig. 3, *p* = 0.4). A similar effect was observed among those with early-stage (I and II) disease, with no statistically significant difference found in overall survival (Supplementary Fig. 5, *p* = 0.11). Variations in survival occurred by subsite, with the greatest survival rates occurring among patients with HPV-positive cancers of the oropharynx (Supplementary Figs. 6 and 7, *p* < 0.0001).

Kaplan-Meier log-rank test analysis detected large differences in survival with declines in ECOG performance status (Supplementary Fig. 8, *p* < 0.0001). Large differences in mortality were detected among those who smoked or consumed alcohol to excess (Supplementary Figs. 9 and 10), with those who reported a current or former smoking status or excess alcohol consumption suffering from significantly increased mortality compared with those who never smoked or drank alcohol (*p* < 0.0001, *p* = 0.001). No survival differences in sex were detected in Kaplan-Meier analysis (Supplementary Fig. 11, *p* = 0.86).

**Table 2:** Cox Proportional Hazards Regression Analysis.

	Hazard Ratio	95% Confidence Interval		*p*	Adj. Hazard Ratio	95% Confidence Interval		*p*
		Lower	Upper			Lower	Upper	
Cohort				0.2				
2019	0.88	0.69	1.13		0.94	0.72	1.22	0.63
2020	REF	REF	REF		REF	REF	REF	
2022	0.80	0.61	1.03		0.93	0.70	1.22	0.59
Site				**<0.001**				
Oral Cavity	**2.60**	**1.78**	**3.81**		0.84	0.55	1.29	0.43
Oropharynx HPV-Positive	REF	REF	REF		REF	REF	REF	
Oropharynx HPV-Negative	**4.45**	**2.92**	**6.79**		1.51	0.95	2.42	0.08
Oropharynx HPV Missing	**14.35**	**6.62**	**31.11**		1.22	0.52	2.91	0.65
Larynx	**2.68**	**1.83**	**3.92**		0.95	0.62	1.44	0.80
Nasopharynx	**2.83**	**1.47**	**5.43**		1.78	0.89	3.54	0.10
Hypopharynx	**5.25**	**3.37**	**8.16**		1.59	0.96	2.62	0.07
Cancer of Unknown Primary (CUP)	**3.80**	**2.13**	**6.77**		1.30	0.69	2.46	0.41
Oropharynx ca. HPV Status				**<0.001**				
HPV-Positive	**0.22**	**0.15**	**0.34**		N/A	N/A	N/A	
HPV-Negative	REF	REF	REF		N/A	N/A	N/A	
Missing	**3.26**	**1.52**	**6.99**		N/A	N/A	N/A	
Age at MDT (5-Year increase)	**1.24**	**1.18**	**1.30**	**<0.001**	**1.14**	**1.07**	**1.21**	**<0.001**
Sex				0.9				
Male	0.98	0.78	1.23		N/A	N/A	N/A	
Female	REF	REF	REF		N/A	N/A	N/A	
SIMD Quintile				**0.03**				
1 (20% Most Deprived)	**1.87**	**1.23**	**2.85**		1.04	0.66	1.62	0.87
2	**1.78**	**1.14**	**2.78**		0.94	0.59	1.50	0.80
3	1.42	0.88	2.29		0.79	0.48	1.30	0.36
4	1.48	0.90	2.42		1.11	0.67	1.83	0.69
5 (20% Least Deprived)	REF	REF	REF		REF	REF	REF	
Disease				**<0.001**				
Early	REF	REF	REF		N/A	N/A	N/A	
Advanced	**4.10**	**3.16**	**5.30**		N/A	N/A	N/A	
Missing	**60.17**	**21.58**	**167.80**		N/A	N/A	N/A	
AJCC Stage				**<0.001**				
I	REF	REF	REF		REF	REF	REF	
II	**2.16**	**1.36**	**3.42**		**1.79**	**1.12**	**2.87**	**0.01**
III	**3.90**	**2.60**	**5.83**		**2.87**	**1.91**	**4.33**	**<0.001**
IV (IV, IVa, IVb & IVc)	**7.06**	**4.90**	**10.15**		**4.42**	**2.99**	**6.55**	**<0.001**
Missing	**87.15**	**30.34**	**250.34**		**160.28**	**36.69**	**700.14**	**<0.001**
Smoking history				**<0.001**				
Never	REF	REF	REF		REF	REF	REF	
Ever	**1.85**	**1.40**	**2.44**		1.26	0.92	1.72	0.15
Missing	**10.09**	**1.39**	**73.31**		**0.05**	**0.00**	**0.63**	**0.02**
History of alcohol excess				**0.001**				
Never	REF	REF	REF		REF	REF	REF	
Ever	**1.42**	**1.15**	**1.75**		1.13	0.89	1.44	0.32
Missing	7.01	0.98	50.29		NA	NA	NA	NA
ECOG PS				**<0.001**				
0	REF	REF	REF		REF	REF	REF	
1	**2.64**	**2.02**	**3.44**		**1.84**	**1.38**	**2.45**	**<0.001**
2	**5.62**	**4.17**	**7.57**		**3.63**	**2.60**	**5.07**	**<0.001**
3	**12.00**	**8.48**	**16.97**		**6.99**	**4.63**	**10.54**	**<0.001**
4	**45.23**	**20.19**	**101.30**		**21.00**	**8.82**	**49.99**	**<0.001**
Emergency Tracheostomy				**0.01**				
No	REF	REF	REF		N/A	N/A	N/A	
Yes	**1.98**	**1.23**	**3.18**		N/A	N/A	N/A	
Treatment Intent				**<0.001**	N/A	N/A	N/A	
Curative	**0.10**	**0.08**	**0.12**		N/A	N/A	N/A	
Palliative	REF	REF	REF		N/A	N/A	N/A	
N/A	N/A	N/A	N/A		N/A	N/A	N/A	
Treatment Delivered				**<0.001**				
BSC	REF	REF	REF		N/A	N/A	N/A	
Palliative SACT	**0.46**	**0.32**	**0.65**		N/A	N/A	N/A	
RT alone	**0.07**	**0.05**	**0.10**		N/A	N/A	N/A	
CRT	**0.03**	**0.02**	**0.04**		N/A	N/A	N/A	
Surgery alone	**0.06**	**0.04**	**0.09**		N/A	N/A	N/A	
Surgery + CRT	**0.07**	**0.04**	**0.14**		N/A	N/A	N/A	
Surgery + RT	**0.06**	**0.03**	**0.09**		N/A	N/A	N/A	

Significance highlighted in **bold**.

*N.A.* Not applicable assessed, *SIMD Quintile* Scottish index of Multiple Deprivation Quintile, *ECOG PS* Performance Status (0 = fully active; 1 = Restricted in physically strenuous activity but ambulatory; 2 = Ambulatory and capable of all selfcare but unable to carry out any work activities; 3 = Capable of only limited selfcare; confined to bed or chair more than 50% of waking hours; 4 = Completely disabled; cannot perform any selfcare), *BSC* Best Supportive Care, *CRT* Chemo-radiotherapy, *RT* Radiotherapy, *SACT* Systemic Anti-cancer Therapy.

Model adjusted for Age, Subsite, SIMD, AJCC Stage, Smoking status, Alcohol excess, and ECOG-PS.

### Cox regression (Table 2)

Survival was not influenced by year of diagnosis (*p* = 0.20); compared with the 2020 cohort, patients diagnosed in 2019 had a hazard ratio (HR) of 0.88 (95% CI = 0.69–1.13), and those diagnosed in 2022 had an HR of 0.80 (95% CI = 0.61–1.03). After adjustment for confounders (age, subsite, AJCC stage (I-IV), SIMD, smoking and alcohol behaviours, and ECOG-PS), the associations between cohort year and survival remained non-significant with adjusted hazard ratios (aHR) of 0.94 (95% CI = 0.72–1.22) and 0.93 (95% CI = 0.70–1.22) for 2019 and 2022, respectively.

Univariably, survival was significantly influenced by cancer subsite (*p* < 0.001). With reference to patients with HPV-positive OPC who had the most favourable survival, OCC patients had an increased mortality risk with an unadjusted HR of 2.60 (95% CI = 1.78–3.81). Similarly, patients with cancers of the larynx (HR = 2.68, 95% CI = 1.83–3.92), nasopharynx (HR = 2.83, 95% CI = 1.47–5.43), HPV-negative oropharynx (HR = 4.45, 95% CI = 2.92–6.97), unknown primary (HR = 3.80, 95% CI = 2.13–6.77) and the hypopharynx (HR = 5.25, 95% CI = 3.37–8.16) had comparitively and increasingly deleterious outcomes when compared with HPV-positive OPC. The poorest survival outcomes were observed among patients with OPC and missing HPV data (HR = 14.35, 95% CI = 6.62–31.11).

Upon adjustment of the Cox regression model, all subsite associations were attenuated and lost statistical significance. Among OPC cases, HPV status was strongly associated with survival (*p* < 0.001). Patients with HPV-positive tumours exhibited significantly improved survival rates (HR = 0.22, 95% CI = 0.15–0.34) when compared with OPC patients with HPV negative tumours. OPC patients with a missing HPV status had markedly worse survival (HR = 3.26, 95% CI = 1.52–6.99).

An increased age at diagnosis was significantly associated with increased mortality (aHR for an increase in five years of age = 1.14, 95% CI = 1.07–1.21). No significant differences in survival were found when evaluating by sex (*p* = 0.90). Socioeconomic deprivation was strongly associated with increased mortality (*p* = 0.03). Patients from SIMD-1 (the 20% most deprived) areas had an increased HR of 1.87 (95% CI = 1.23–2.85) compared with those from SIMD 5. However, upon adjustment, socioeconomic status was not a significant predictor of mortality in the multivariable model.

Patients’ stage of disease was highly predictive of survival. Relative to early-stage disease, advanced stage disease (III and IV) was associated with a four-fold increase in mortality (HR = 4.10, 95% CI = 3.16–5.30). This remained upon adjustment, with an increasing AJCC stage significantly associated with an increased mortality (Stage II: aHR = 1.79, 95% CI = 1.12–2.87; Stage III: aHR = 2.87, 95% CI = 1.91–4.33; Stage IV: aHR = 4.42, 95% CI = 2.99–6.55). Similarly, patients’ functional status was also strongly predictive of survival outcomes (*p* < 0.001). A poorer ECOG PS was strongly and independently linked with increases in mortality (ECOG PS1: aHR = 1.84, 95% CI = 1.38–2.45; ECOG PS2: aHR = 3.63, 95% CI = 2.60–5.07; ECOG PS3: aHR = 6.99, 95% CI = 4.63–10.64).

Patients with a history of smoking or alcohol excess were found to have increased mortality (ever-smoker: HR = 1.85, 95% CI = 1.40–2.44, ever-alcohol excess: HR = 1.42, 95% CI = 1.15–1.75). However, these behaviours lost statistical significance upon multivariable adjustment.

Curative treatment intent at MDT was associated with significantly improved survival (*p* < 0.001). Although informed by site and stage, treatment modality was associated with survival; those receiving surgical, oncological or combination approaches exhibited the best survival outcomes (*p* < 0.001).

**Table 3:** Logistic Regression Analysis Modelling for Advanced-stage Disease (AJCC stages III and IV).

	Odds Ratio	95% Confidence Interval		*p*	Adj. Odds Ratio	95% Confidence Interval		*p*
		Lower	Upper			Lower	Upper	
Cohort								
2019	**0.62**	**0.43**	**0.91**	**0.01**	**0.54**	**0.35**	**0.84**	**<0.01**
2020	REF	REF	REF		REF	REF	REF	
2022	**0.61**	**0.42**	**0.88**	**0.01**	**0.63**	**0.40**	**0.96**	**0.03**
Site								
Oral Cavity	**4.63**	**2.93**	**7.45**	**<0.001**	**2.70**	**1.62**	**4.56**	**<0.001**
Oropharynx HPV-Positive	REF	REF	REF		REF	REF	REF	
Oropharynx HPV-Negative	**9.28**	**4.94**	**18.20**	**<0.001**	**5.49**	**2.74**	**11.40**	**<0.001**
Oropharynx HPV Missing	**8.02**	**1.77**	**56.30**	**0.01**	**1.99**	**0.32**	**17.10**	**0.48**
Larynx	**4.68**	**2.93**	**7.60**	**<0.01**	**3.37**	**2.01**	**5.74**	**<0.001**
Nasopharynx	**6.24**	**2.33**	**18.70**	**<0.001**	**6.88**	**2.43**	**21.60**	**<0.001**
Hypopharynx	**14.60**	**6.83**	**34.20**	**<0.001**	**10.20**	**4.52**	**25.10**	**<0.001**
Cancer of Unknown Primary (CUP)	**16.00**	**5.78**	**57.20**	**<0.001**	**13.30**	**4.57**	**49.00**	**<0.001**
Oropharynx ca. HPV Status								
HPV-Positive	**0.11**	**0.05**	**0.20**	**<0.001**	N/A	N/A	N/A	
HPV-Negative	REF	REF	REF		N/A	N/A	N/A	
Missing	0.86	0.18	6.26	0.87	N/A	N/A	N/A	
Age at MDT (5-Year)	**1.11**	**1.04**	**1.18**	**<0.01**	0.95	0.87	1.03	0.20
Sex								
Male	1.15	0.83	1.61	0.40	N/A	N/A	N/A	
Female	REF	REF	REF		N/A	N/A	N/A	
SIMD Quintile								
1 (20% Most Deprived)	**1.95**	**1.15**	**3.31**	**0.01**	1.04	0.56	1.94	0.91
2	**1.81**	**1.03**	**3.22**	**0.04**	1.01	0.52	1.98	0.97
3	1.74	0.96	3.17	0.07	1.36	0.68	2.72	0.38
4	1.27	0.68	2.37	0.45	1.17	0.57	2.40	0.66
5 (20% Least Deprived)	REF	REF	REF		REF	REF	REF	
Smoking history								
Never	REF	REF	REF		REF	REF	REF	
Ever	**2.29**	**1.61**	**3.26**	**<0.001**	**1.44**	**0.93**	**2.22**	0.10
Missing	N/A	N/A	N/A		NA	NA	NA	
History of alcohol excess								
Never	REF	REF	REF		REF	REF	REF	
Ever	**2.09**	**1.50**	**2.93**	**<0.001**	**1.53**	**1.02**	**2.30**	**0.04**
Missing	N/A	N/A	N/A		N/A	N/A	N/A	
ECOG PS								
0	REF	REF	REF		REF	REF	REF	
1	**2.55**	**1.78**	**3.66**	**<0.001**	**2.32**	**1.54**	**3.54**	**<0.001**
2	**4.68**	**2.81**	**8.10**	**<0.001**	**3.96**	**2.20**	**7.38**	**<0.001**
3	**12.30**	**5.24**	**36.00**	**<0.001**	**13.10**	**5.08**	**41.40**	**<0.001**
4	3.07	0.65	21.70	0.18	2.25	0.38	19.00	0.40
Emergency Tracheostomy								
No	REF	REF	REF		N/A	N/A	N/A	
Yes	**6.83**	**1.97**	**43.00**	**0.01**	N/A	N/A	N/A	
Treatment Intent					N/A	N/A	N/A	
Curative	**0.11**	**0.07**	**0.17**	**<0.001**	N/A	N/A	N/A	
Palliative	REF	REF	REF		N/A	N/A	N/A	
N/A	N/A	N/A	N/A		N/A	N/A	N/A	
Treatment Delivered								
BSC	N/A	N/A	N/A		N/A	N/A	N/A	
Palliative SACT	N/A	N/A	N/A		N/A	N/A	N/A	
RT alone	N/A	N/A	N/A		N/A	N/A	N/A	
CRT	N/A	N/A	N/A		N/A	N/A	N/A	
Surgery alone	N/A	N/A	N/A		N/A	N/A	N/A	
Surgery + RT	N/A	N/A	N/A		N/A	N/A	N/A	
Surgery + CRT	N/A	N/A	N/A		N/A	N/A	N/A	

Model Significance highlighted in **bold**.

*N.A.* Not applicable/assessed, *SIMD Quintile* Scottish index of Multiple Deprivation Quintile.

*ECOG PS* Performance Status (0 = fully active; 1 = Restricted in physically strenuous activity but ambulatory; 2 = Ambulatory and capable of all selfcare but unable to carry out any work activities; 3 = Capable of only limited selfcare; confined to bed or chair more than 50% of waking hours; 4 = Completely disabled; cannot perform any selfcare).

*BSC* Best Supportive Care, *CRT* Chemo-radiotherapy, *RT* Radiotherapy, *SACT* Systemic Anti-cancer Therapy.

Model adjusted for Age, Subsite, SIMD, Smoking status, Alcohol excess and ECOG-PS.

### Stage (Table 3)

Logistic regression analysis modelling the crude and adjusted (age, subsite, SIMD, ECOG PS, alcohol and smoking behaviours) odds of advanced-stage disease (III and IV). The cohort year was strongly associated with advanced-stage disease. When compared with those in the 2020 cohort, people with HNSCC in the 2019 and 2022 cohorts were signficantly less likely to present with a advanced-stage cancers (2019: Adj. OR = 0.54, 95% CI = 0.35–0.84, 2020: Adj. OR = 0.63, 95% CI = 0.40–0.96).

An increasing age at MDT was initially associated with advanced-stage disease, but upon adjustment, this lost statistical significance (aOR 5-year increase: 0.95; 95% CI: 0.87–1.03). A similar effect was observed among those from more socioeconomically deprived SIMD-quintile areas; however, these associations were attenuated in the multivariable model. People who reported a history of tobacco smoking had increased odds of advanced-stage disease, but this lost significance upon further adjustment (OR: 1.44; 95% CI: 0.93–2.22). A similar effect was observed among those with a history of excess alcohol consumption (aOR: 1.53; 95% CI: 1.02–2.30).

When compared with HPV-positive cancers of the oropharynx, significantly increased odds of advanced-stage disease were seen among cancers of the oral cavity (aOR: 2.70; 95% CI: 1.62–4.56), HPV-negative oropharynx cases (aOR: 5.49; 95% CI: 2.74–11.40) and larynx (aOR: 3.37; 95% CI: 2.01–5.74). Strong associations were also observed for cancers of the hypopharynx, nasopharynx, and unknown primaries. A decline in performance status was highly and independently associated with higher stage cancers. Compared with performance status 0, those with ECOG PS 1, 2, and 3 all had greater adjusted odds of advanced stage disease presentation (*p* < 0.001).

## Discussion

The COVID-19 pandemic posed great challenges and disruptions to healthcare in Scotland, and beyond, with projections that pandemic-mediated disruptions to healthcare would result in a major up-staging of cancer cases and declines in survival[8]. This study identified an increased burden of advanced-stage disease in the 2020 cohort, accompanied by shifts in presentation by subsite and socioeconomic distributions. Despite changes in the stage and socioeconomic deprivation characteristics, the overall survival of patients among the cohorts was found to be comparable (*p* = 0.22). Although a reduced Hazard ratio was observed for years 2019 relative to 2020, this remained non-significant and was further attenuated following adjustment for potential confounders. Similarly, no significant differences in MDT decisions (curative vs palliative) or treatment modality delivered were observed over the study period.

The existing literature examining how the COVID-19 pandemic influenced HNSCC survival is currently limited in quantity and scope. Studies by Bloom et al. and Venkatasai et al. identified no differences in short-term survival outcomes, albeit among single-centre oncology cohorts with smaller sample sizes in the USA and India, respectively[19, 20]. One population-based study by Peacock et al. identified a modest, but non-significant, decline in one-year survival among patients recorded in the Belgian national cancer registry [21], while a larger single-site study by León et al. found no difference in short-term survival outcomes among patients at a large tertiary centre in Spain[22]. These studies also observed reduced numbers of incident cases during 2020, in contrast with the consistent numbers observed in this study and at a national level in Scotland[23]. Our study offers a valuable addition to the limited quantity of existing literature, suggesting that, reassuringly, the COVID-19 pandemic has not affected survival outcomes. This contrasts with wider literature suggestive of declines in the survival of many cancers associated with the COVID-19 pandemic[24].

A significant increase in the proportion of HNSCCs diagnosed at advanced stages (III and IV) was observed among the 2020 cohort. Although strongly associated with survival outcomes, the increased burden of advanced-stage disease did not ultimately translate to significant differences in survival over the study period. A possible explanation for this may lie in the prognostic accuracy of contemporary staging systems - although TNM-8 staging has been shown to be an improvement upon its predecessor, it has been shown to vary in discriminative accuracy when assessing mortality risk[25]. Further changes in the staging of HPV-related OPSCC are planned to be implemented in 2026 in the UICC TNM 9th edition to allow for improved prognostic discrimination[26]. Given that a large proportion of our cohort comprises HPV-related OPSCC it is possible that sub-optimal prognostic accuracy is demonstrated here by the increased burden of advanced-stage disease but unchanged survival outcomes. It is also feasible that survival differences may become apparent with increased follow up, or indeed that small differences in survival have not been detected by our modest sized cohorts. Interestingly, recent data has demonstrated that mRNA vaccines against COVID-19 confer improved survival among patients with advanced-stage non-small-cell lung cancer or melanoma receiving immune-checkpoint inhibitors owing to activation of systemic immunity, potentiating antitumour responses. While no such data exists in HNSCC or for other anti-cancer treatment modalities, one could hypothesise that a similar phenomenon may be responsible for the better than anticipated survival in the 2020 cohort and is worthy of further investigation[27].

Moreover, the majority of HNSCC cases were diagnosed at an advanced stage across all three cohorts in this study. It may be that the detrimental survival effects of an increased advanced stage disease burden in 2020 were shrouded by the overall burden of advanced-stage disease in the study. Unfortunately, the burden reported is consistent with the existing literature on the typical advanced-stage presentation of HNSCC, particularly in the UK[28]. This is a well-known phenomenon observed with HNSCCs and is a key prognostic determinant[29]. Similarly, where patients had missing HPV status data, this was often a poor prognostic marker. These patients typically presented with such advanced disease and poor comorbid status that conducting a biopsy to confirm a clinical diagnosis was inappropriate.

Much like the literature surrounding survival and treatment modalities during the COVID-19 pandemic, there is some variation in the reporting of how COVID-19 affected the stage of presentation of HNSCCs. Our findings of an increased burden of advanced-stage disease contrast with a previous west of Scotland analysis but are likely attributable to the differing time periods chosen in these studies[30]. A rapid review and meta-analysis by Clements et al. identified 16 studies that reported no differences in stage of HNSCC diagnosed before and during the pandemic; however, the meta-analysis conducted in this study revealed a 17% increased shift towards advanced-stage disease among those diagnosed during the COVID-19 pandemic[9]. Our study results were consistent with this finding, but reassuringly, did ultimately not translate to detrimental survival outcomes.

In our study, no differences in treatment intent nor delivery were shown. Modest but non-significant changes in combination surgical approaches were observed in 2020 and may be explained by the increased proportion of OCC cases described. These findings are consistent with analyses of other Scottish cancer centres, where HNSCC treatments were consistently delivered in spite of the pandemic and without increased rates of COVID infection[6, 31]. Other studies also detected no significant differences in treatment modality delivery to HNSCC patients during the COVID-19 pandemic when compared with pre-pandemic data, although mainly from smaller, single-site studies[32–34]. In contrast, studies have observed changes in treatment modalities delivered, particularly declines in the number of surgical treatments delivered[35, 36].

A systematic review by Grumstrup et al. found no difference in treatment modality delivered to HNSCC patients among six studies (primarily from Europe and North America), which reported treatment modalities comparing COVID and non-COVID cohorts[37]. Changes in treatment delivery may have been attributable to pandemic-related changes in treatment protocols. For example, the work by Martelli et al. hypothesised that the substantial increases in oncological therapies and decline in surgical treatments delivered during the COVID-19 pandemic in Brazil were due to national shifts in treatment pathways[38]. Ultimately, our study suggests that, reassuringly, the management of patients in the west of Scotland remained comparable despite the challenges the COVID-19 pandemic posed.

Changes in the subsite characteristics of patients who presented to the MDT were observed among the 2020 cohort. An increase in the proportion of OCC cases occurred, accompanied by a decline in the proportion of larynx cancers. However, the subsite distribution of cancer cases among the 2022 cohort reverted to a pattern more akin to the pre-pandemic (2019) cohort. More widely, the patterns were consistent with HNSCC incidence recorded by the national cancer registry, including the increase in the number of OCC cases and concurrent decline in the number of larynx cancer cases. Stable numbers of cases were observed among our cohorts, as reflected in the cancer registry data, although the registry did observe an increased number of total incident cases in 2021 and 2022[39]. Comparable case numbers might also be attributable to additional remote triaging and risk calculator measures implemented during the pandemic[40].

The exact mechanisms underlying the changed numbers of OCC, CUPs and larynx cancers recorded among the cohorts are unclear, although there are several potential hypotheses. A large proportion of OCCs are referred by general dental practitioners; strict COVID-19 measures and closures were enforced on general dental practices until Phase 2 of public health relaxation measures as of the 22nd of June[41, 42]. The increases in OCC observed during the 2020 cohort period may be attributable to the initial closure of dental practices and associated delays in referrals.

Similarly, the shift to remote consultations in general medical practice may have also led to diagnostic challenges explaining changes in the proportions of cancers diagnosed[43]. The reduced numbers of larynx cancers could also be attributable to overlap with COVID-19 symptoms and shielding behaviours among those with existing smoking-related comorbidities, e.g., COPD. More widely, declines in larynx cancer rates have been observed in high-income countries, including Scotland, which is in keeping with declining smoking trends in the population[1]. Although some studies do report similar subsite changes over the pandemic years, many of these were single sites or institutions, limiting generalisability[44]. Generally, there is little literature evaluating HNSCC subsite incidence across the pre-, intra-, and post-pandemic years, making it challenging to definitively ascertain how the pandemic may have changed subsite trends and presentation.

The presentation of patients across the cohorts was strongly socioeconomically patterned. This is consistent with the existing local and international literature concerning HNSCC incidence, with the highest incidence rates among those most socioeconomically deprived[1, 45]. Notably, a reduction in the proportions of patients from lower SES groups (SIMD-1 quintile) was observed among the 2020 cohort and significant survival inequalities were detected (*p* = 0.03). This finding mirrors other studies, suggestive of widening inequalities in cancer referrals and diagnoses associated with the pandemic[46]. Furthermore, this is also congruent with the wider literature indicative of the COVID-19 pandemic widening existing socioeconomic and health inequalities[47, 48]. Overall survival exhibited a strong socioeconomic gradient (*p* = 0.02), with the poorest survival occurring among patients from the most socioeconomically deprived areas (SIMD-1 and SIMD-2). Again, this study was also consistent with prior literature evaluating socioeconomic trends of HNSCC survival, with those from poorer socioeconomic backgrounds having the most adverse survival outcomes[49]. The strong socioeconomic inequality gradient underlying this study highlights the need for interventions to help identify and better support at-risk groups in socioeconomically deprived areas.

Consistently across the cohorts, many of the patients in this study reported a history of current or previous tobacco smoking and alcohol excess, both of which were strongly associated with advanced-stage disease. A large proportion of OPC patients presented with HPV-positive tumours. Although associated with more favourable treatment responses and survival when compared with patients with HPV-negative OPCs (as seen in Supplementary Figs. 6 and 7), the growing incidence of HPV-positive OPC in high-income countries will still present challenges to healthcare systems[1]. The high prevalence of these major risk determinants among the cohorts emphasises the continued need for local and population-level prevention strategies - for example, further regulation of tobacco and alcohol, in addition to the continuation and careful monitoring of the national HPV vaccination programme.

This analysis ultimately suggests that while the COVID-19 pandemic was associated with an increase in advanced-stage disease and the widening of inequalities, HNSCC patients in Scotland faced comparable survival rates to patients diagnosed and treated before, during, and after the peaks of the pandemic.

### Limitations and strengths

This study had some limitations. Firstly, some of the behavioural data collected may be subject to potential recall biases when reported by patients and subsequently recorded in the clinical notes. The use of SIMD as a measure of socioeconomic deprivation is subject to the assumption that individuals living within postcode data zones are socioeconomically homogenous; however, analysis has shown it is a robust and comparable measure when compared with individual-level metrics[50].

While this study evaluated the overall survival of cohorts diagnosed before, during and after the implementation of COVID-19 public health measures, the longer-term impacts of the COVID-19 pandemic on HNSCC survival may not have been fully realised; the World Health Organisation did not declare an end to the pandemic until the 5^th^ of May 2023, so this analysis does not reflect the survival of fully “post-pandemic” cohorts[14]. Longer-term follow-up should also be conducted. Nonetheless, the “post-pandemic” period chosen coincides with the relaxation of most COVID-19 public health measures in Scotland and is likely to capture any diagnostic or treatment-related delays associated with the COVID-19 pandemic. Finally, while this study’s findings are likely indicative of the effects of the COVID-19 pandemic at a national level, they may have less international generalisability due to global variations in public health measures and structural differences in healthcare systems.

This study also had several other strengths; clinical data and endpoints were reported and confirmed by the regional MDT. This will have improved the consistency and reliability of the data recorded. The use of multiple survival analysis techniques also improves the robustness of this study, allowing for the assessment of confounders, but also censoring and mortality over time. Although it is not yet feasible to capture long-term survival data, it is well documented that the critical survival period where most recurrences or deaths will occur is in the first one to two years post-diagnosis, which this study successfully captures for all cohorts[51]. Similarly, the usage of MDT data and all incident cases will have ensured that there were no selection biases. The large population coverage of the regional MDT (2.5 million, or 45% of the Scottish population) also offers strong generalisability to national survival trends and outcomes in Scotland[10].

## Conclusions

This study has observed that while the COVID-19 pandemic was associated with changes in subsite distribution, widened socioeconomic inequalities and an increase in advanced-stage disease of HNSCC, this has not translated to changes in intent of treatments delivered or survival outcomes in the west of Scotland. Advanced-stage disease presentation and wide socioeconomic inequalities remain important challenges and targets for future interventions.

## Supplementary information

Supplementary information

## Data Availability

The datasets analysed are not publicly available due to the sensitivity of patient and clinical data, but are available on request and NHS Caldicott Guardian ethical approvals. Code available on reasonable request.
