# Supplementary material for: The COVID-19 pandemic has not influenced survival outcomes of head and neck cancer squamous cell carcinomas in the West of Scotland: a retrospective cohort study
Source: BJC Rep. 2026 Mar 4;4:8. doi: 10.1038/s44276-026-00203-3 (PMC12960927; doi:10.1038/s44276-026-00203-3)
Supplement: Supplementary file 1 — Supplementary information [file 44276_2026_203_MOESM1_ESM.docx]

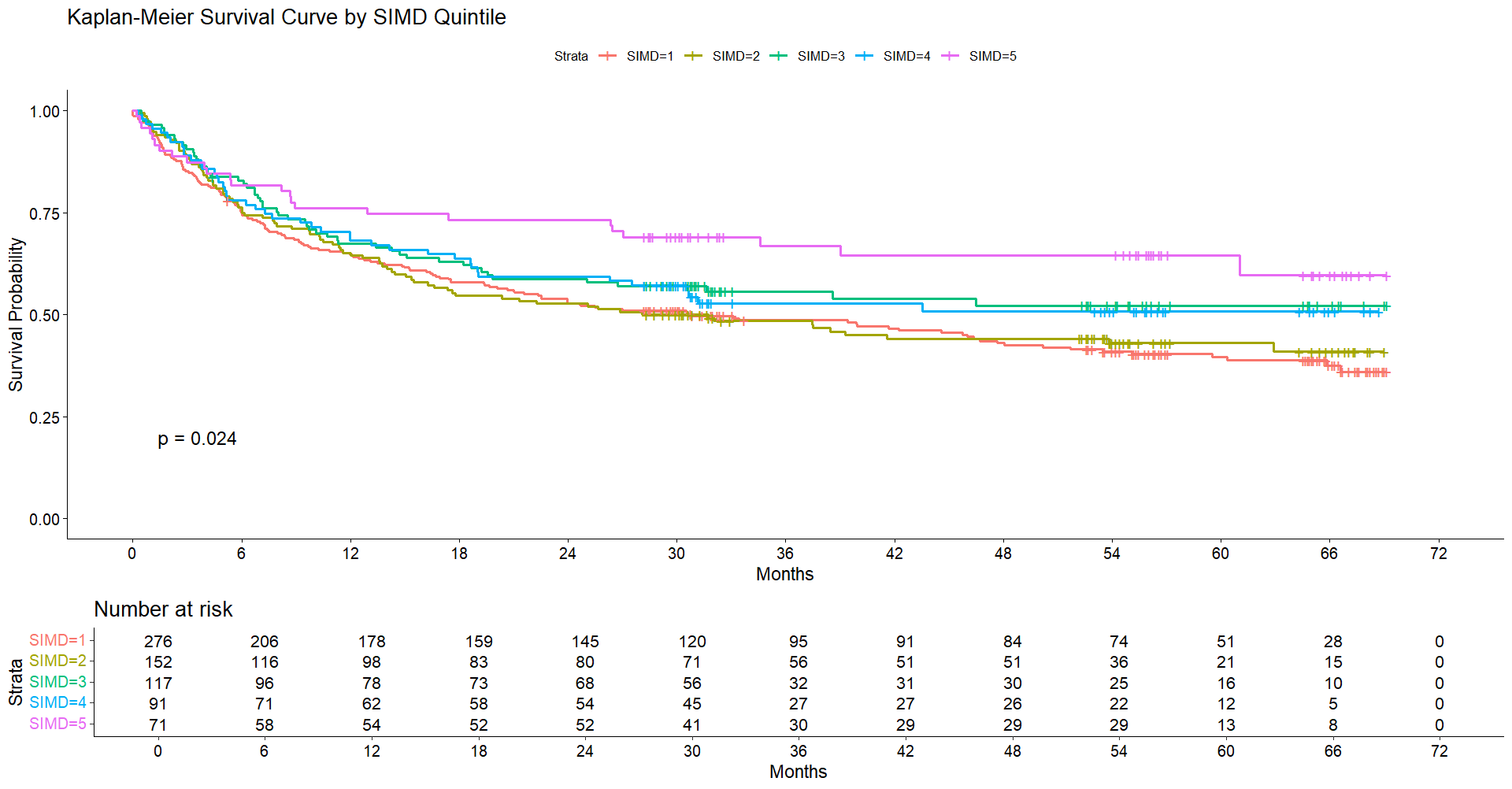


Supplementary Figure 1: Kaplan‐Meier Survival Curve stratified by SIMD (Scottish Index of Multiple Deprivation) for patients diagnosed with Head and Neck Cancer in the west of Scotland among the 2019, 2020 and 2022 cohorts. SIMD 1 = 20% Most deprived, SIMD 5 = 20% Least deprived.


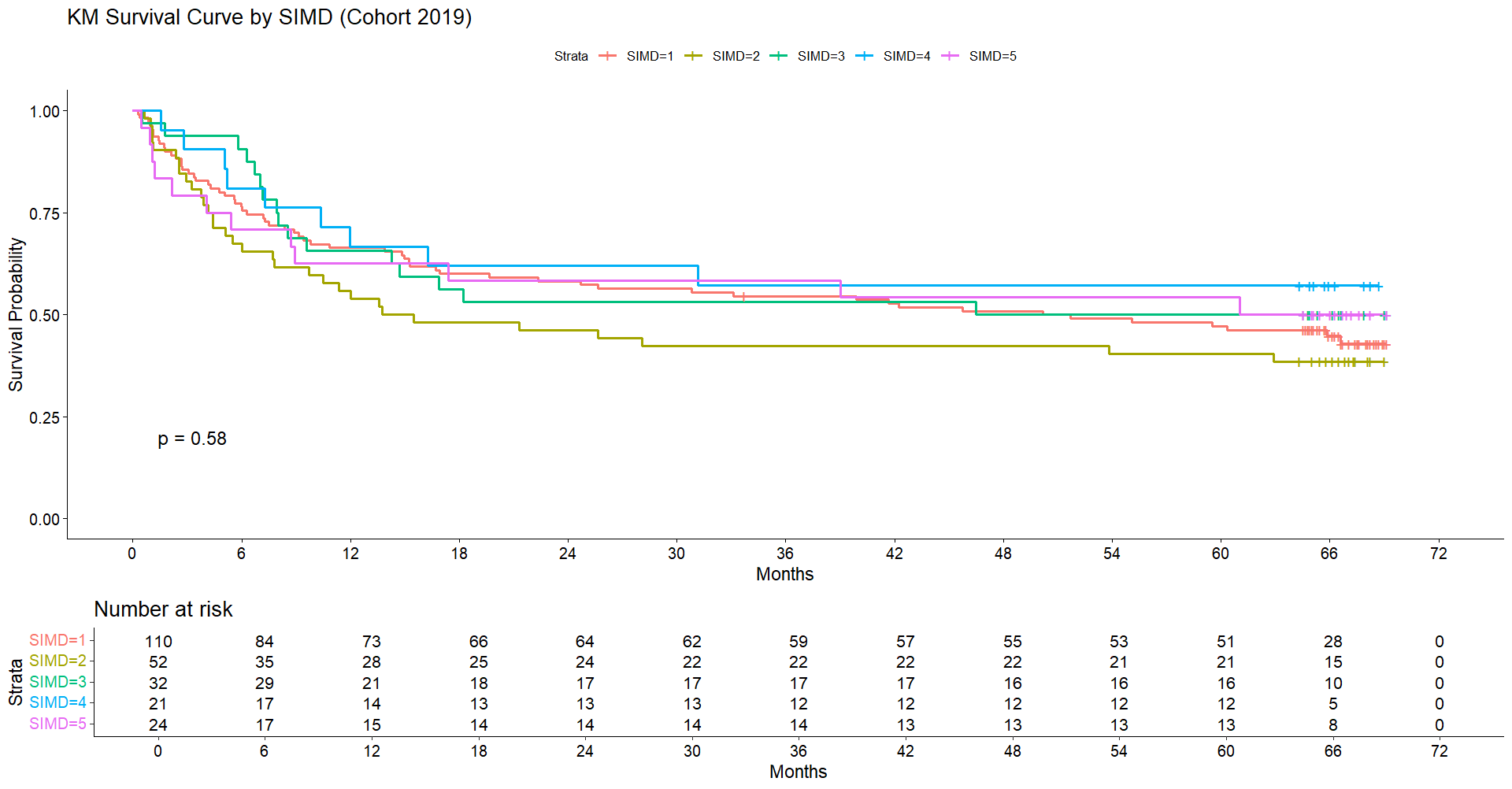


Supplementary Figure 2: Kaplan‐Meier Survival Curve stratified by SIMD (Scottish Index of Multiple Deprivation) for patients diagnosed with Head and Neck Cancer in the west of Scotland among the 2019 cohort. SIMD 1 = 20% Most deprived, SIMD 5 = 20% Least deprived.


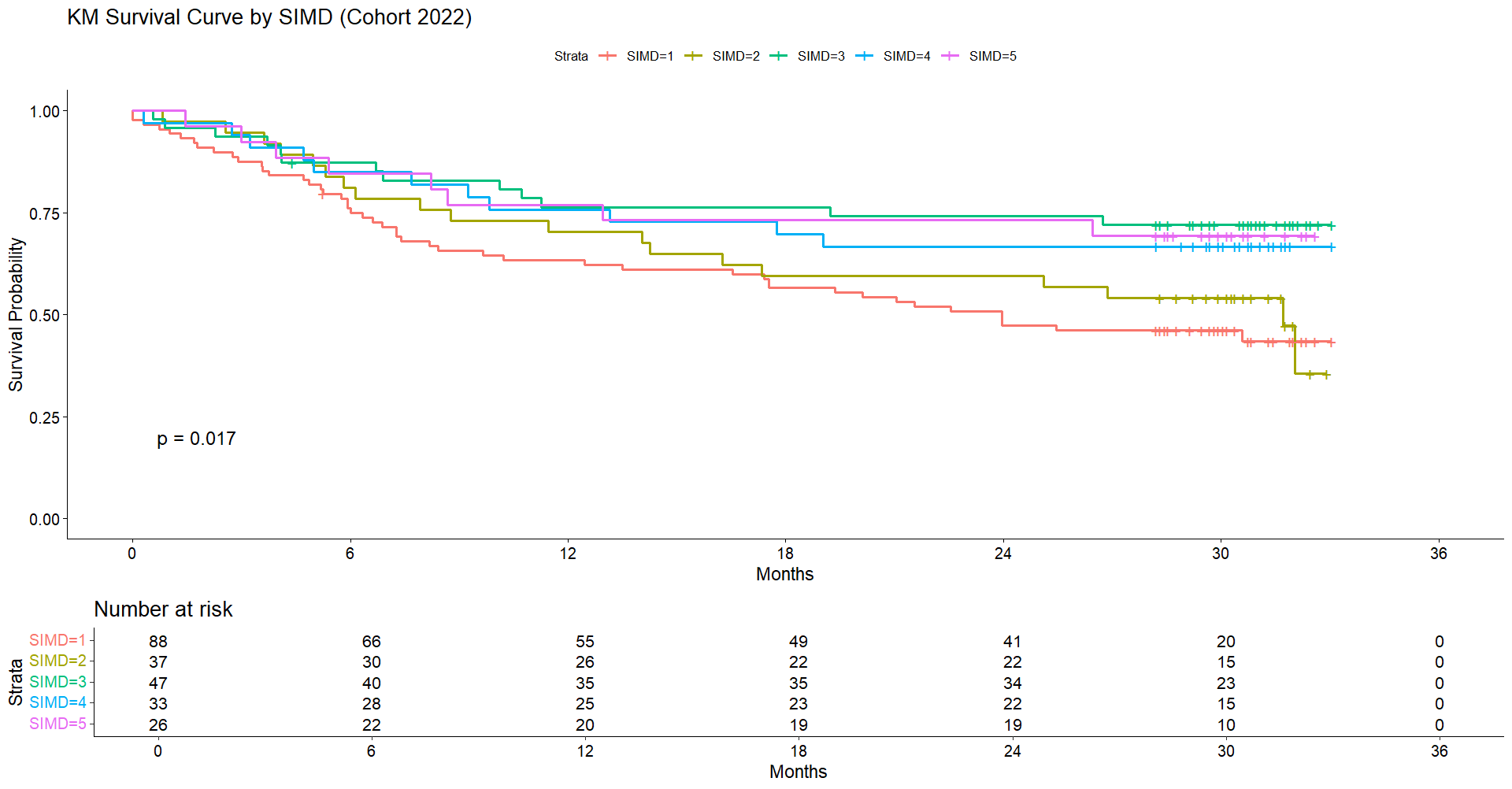


Supplementary Figure 3: Kaplan‐Meier Survival Curve stratified by SIMD (Scottish Index of Multiple Deprivation) for patients diagnosed with Head and Neck Cancer in the west of Scotland among the 2022 cohort. SIMD 1 = 20% Most deprived, SIMD 5 = 20% Least deprived.


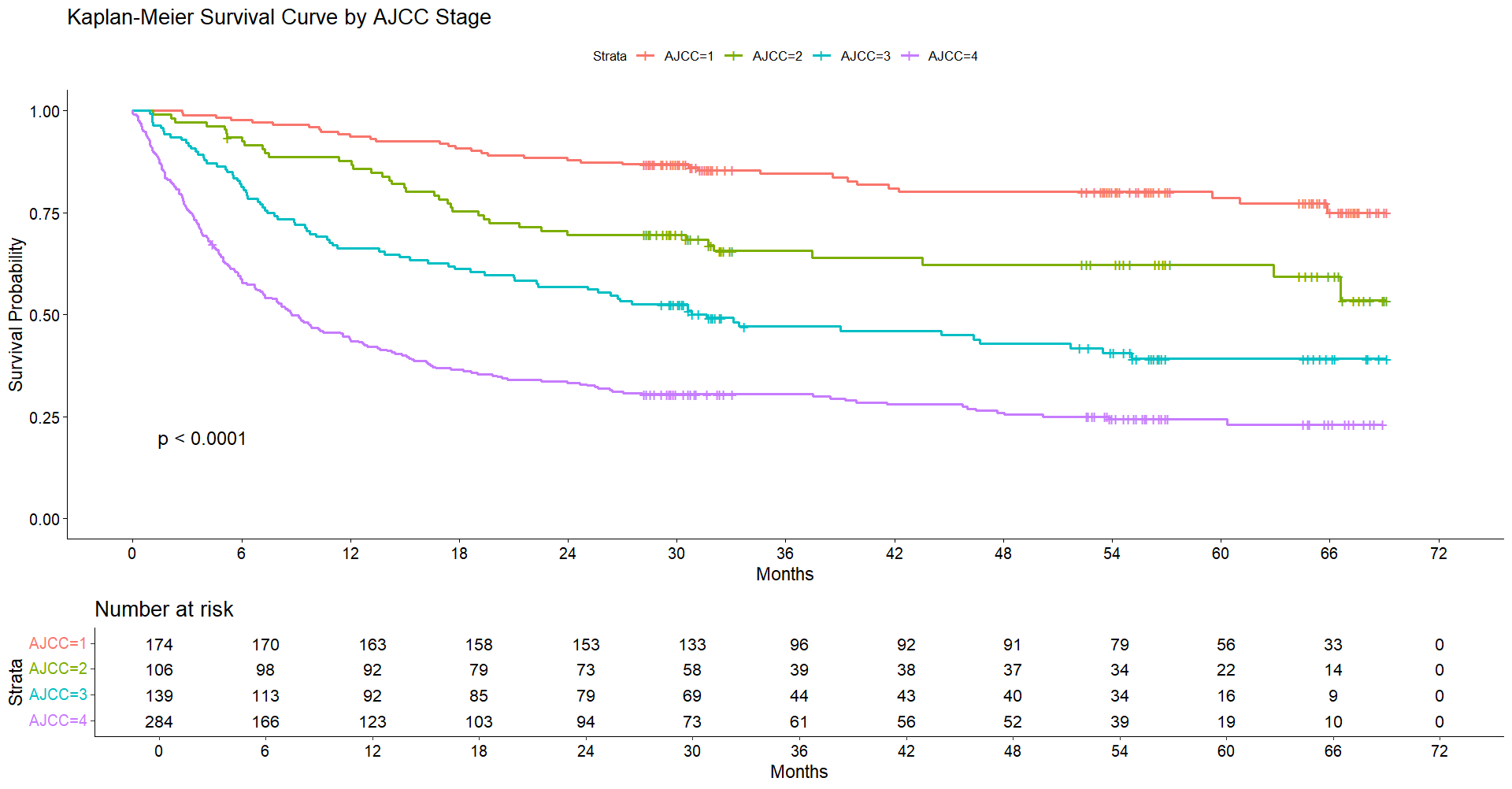


Supplementary Figure 4: Kaplan-Meier Curve by American Joint Committee on Cancer stage of patients in the study (AJCC Stage 4 Includes stages Iva, IVb and IVc), Four patients had missing stage data.


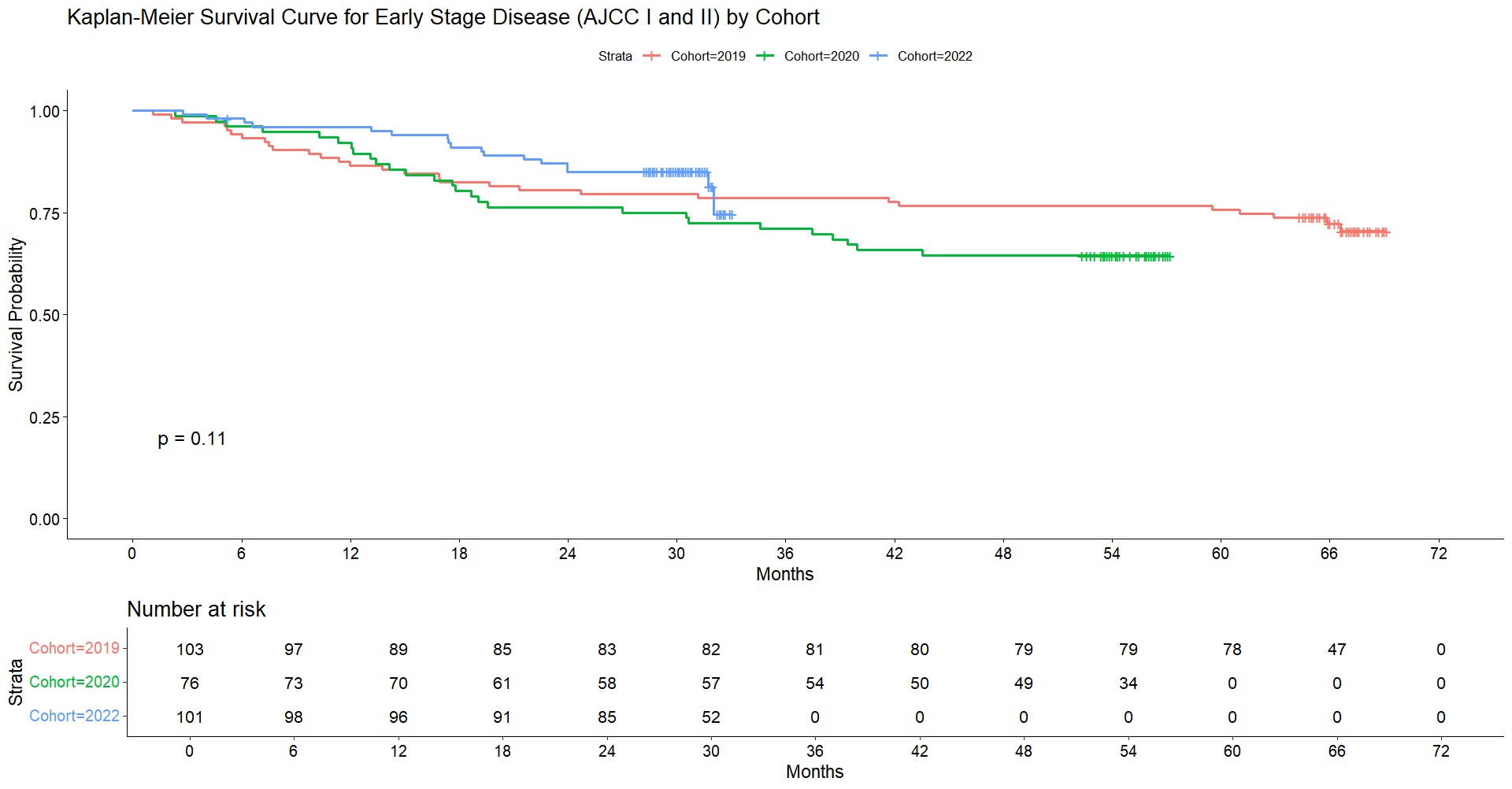


Supplementary Figure 5: Kaplan‐Meier survival curve for patients diagnosed with early stage (AJCC Stage II and II) Head and Neck Cancer in the west of Scotland among the 2019, 2020 and 2022 cohorts.


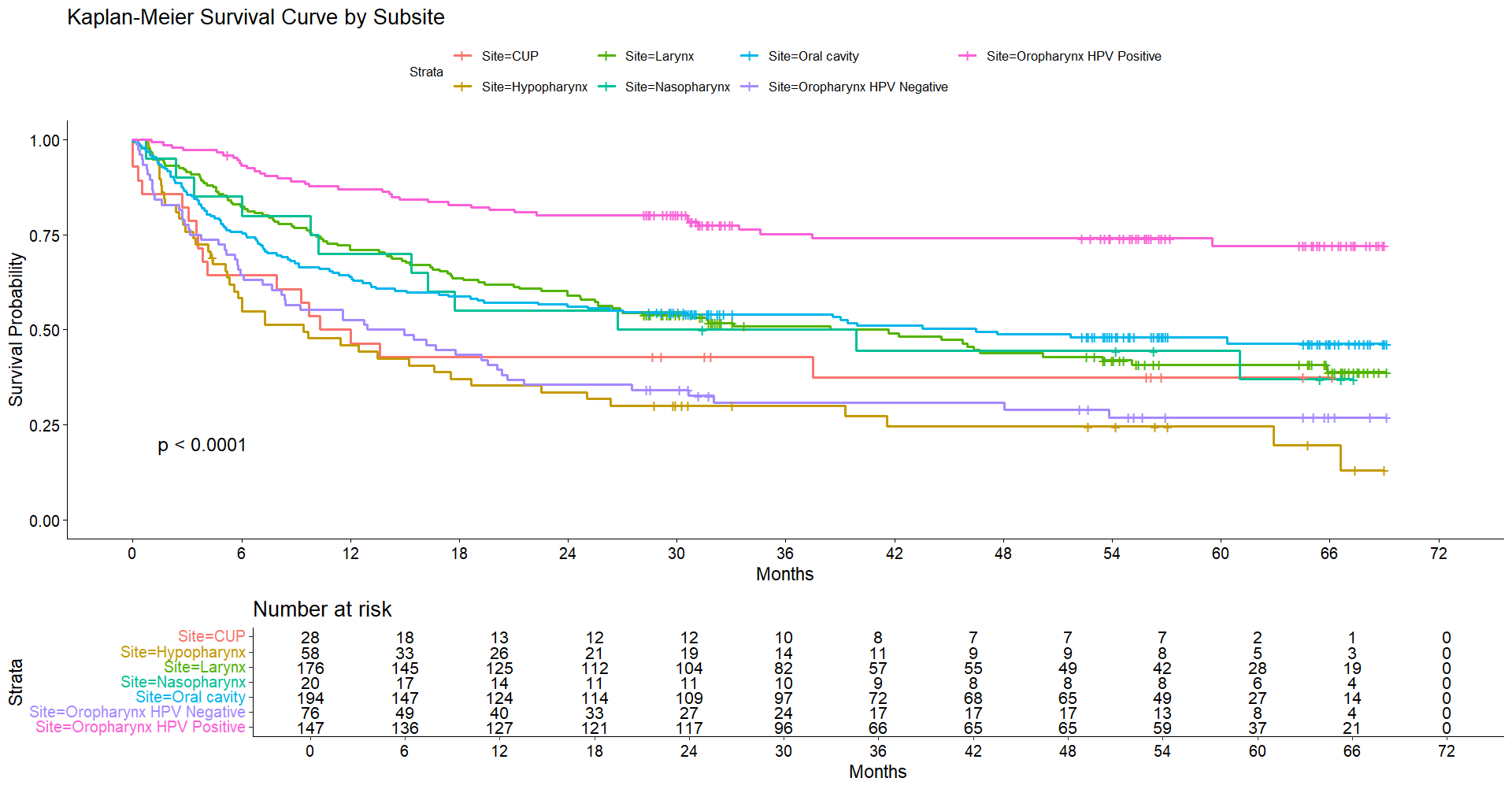
Supplementary Figure 6: Kaplan-Meier Curve by Subsite of HNC patients in the study. CUP = Cancer of Unknown Primary. Four oropharynx cancer patients from the 2019, three from the 2020 and one from the 2022 cohort were unfit to undergo biopsy procedures so lacked P16 status data.


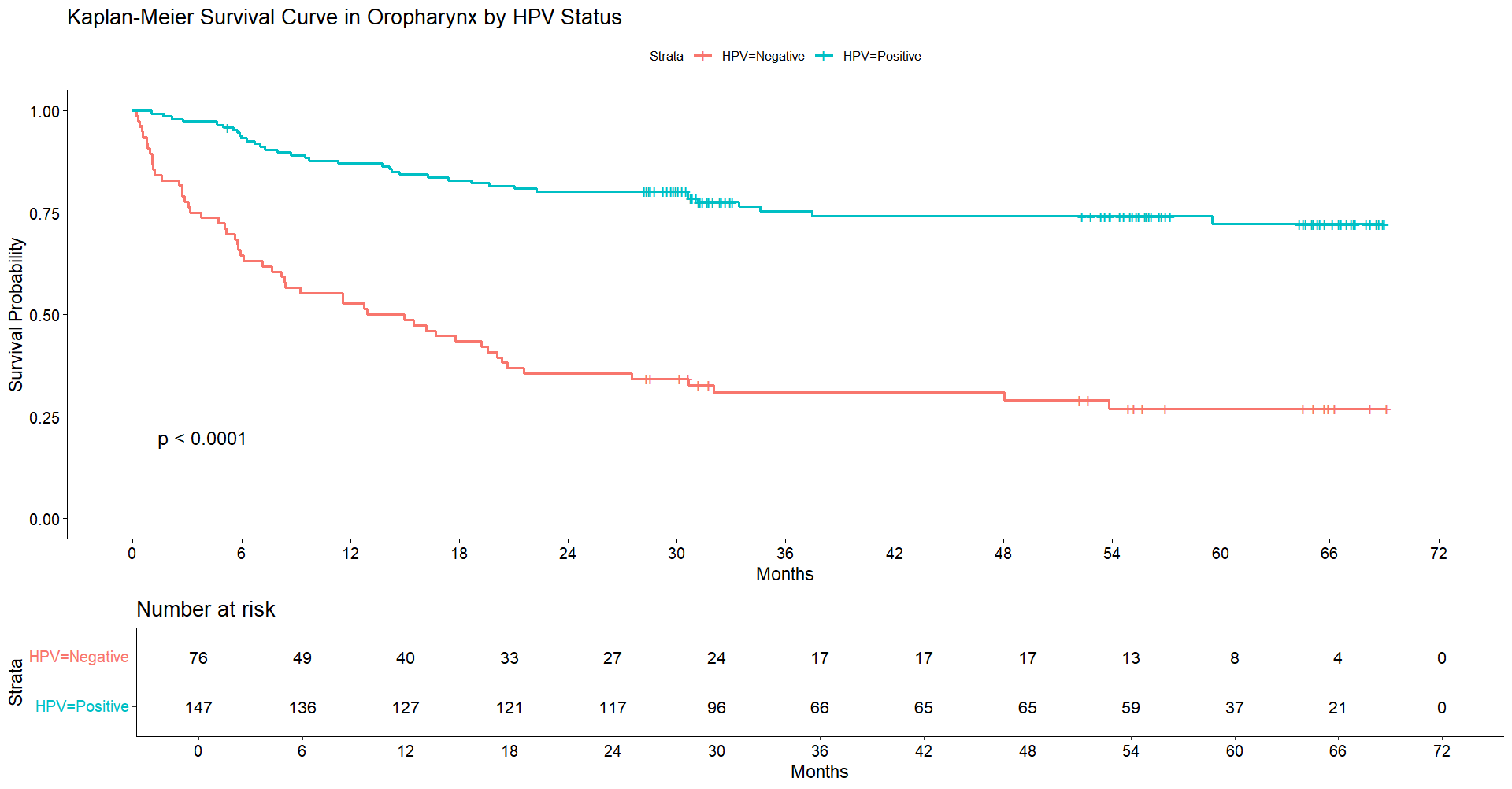


Supplementary Figure 7: Kaplan-Meier Curve by Human Papillomavirus status of patients with oropharynx cancer in the study. Four oropharynx cancer patients from the 2019, three from the 2020 and one from the 2022 cohort were unfit to undergo biopsy procedures so lacked p16 status data.


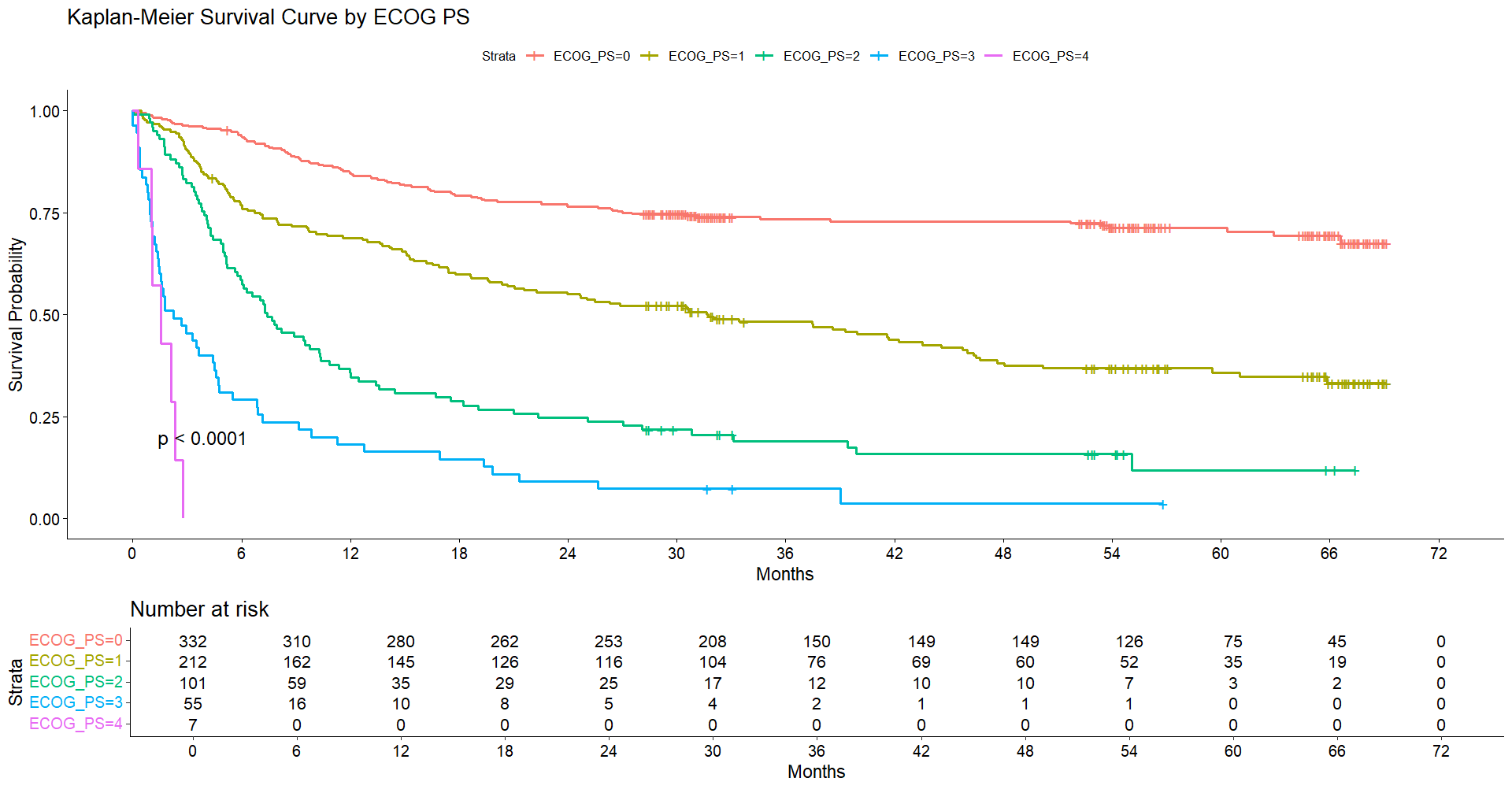


Supplementary Figure 8: Kaplan-Meier Curve by ECOG Performance Status of patients in the study (0 = fully active; 1 = Restricted in physically strenuous activity but ambulatory; 2 = Ambulatory and capable of all selfcare but unable to carry out any work activities; 3 = Capable of only limited selfcare; confined to bed or chair more than 50% of waking hours; 4 = Completely disabled; cannot perform any selfcare).


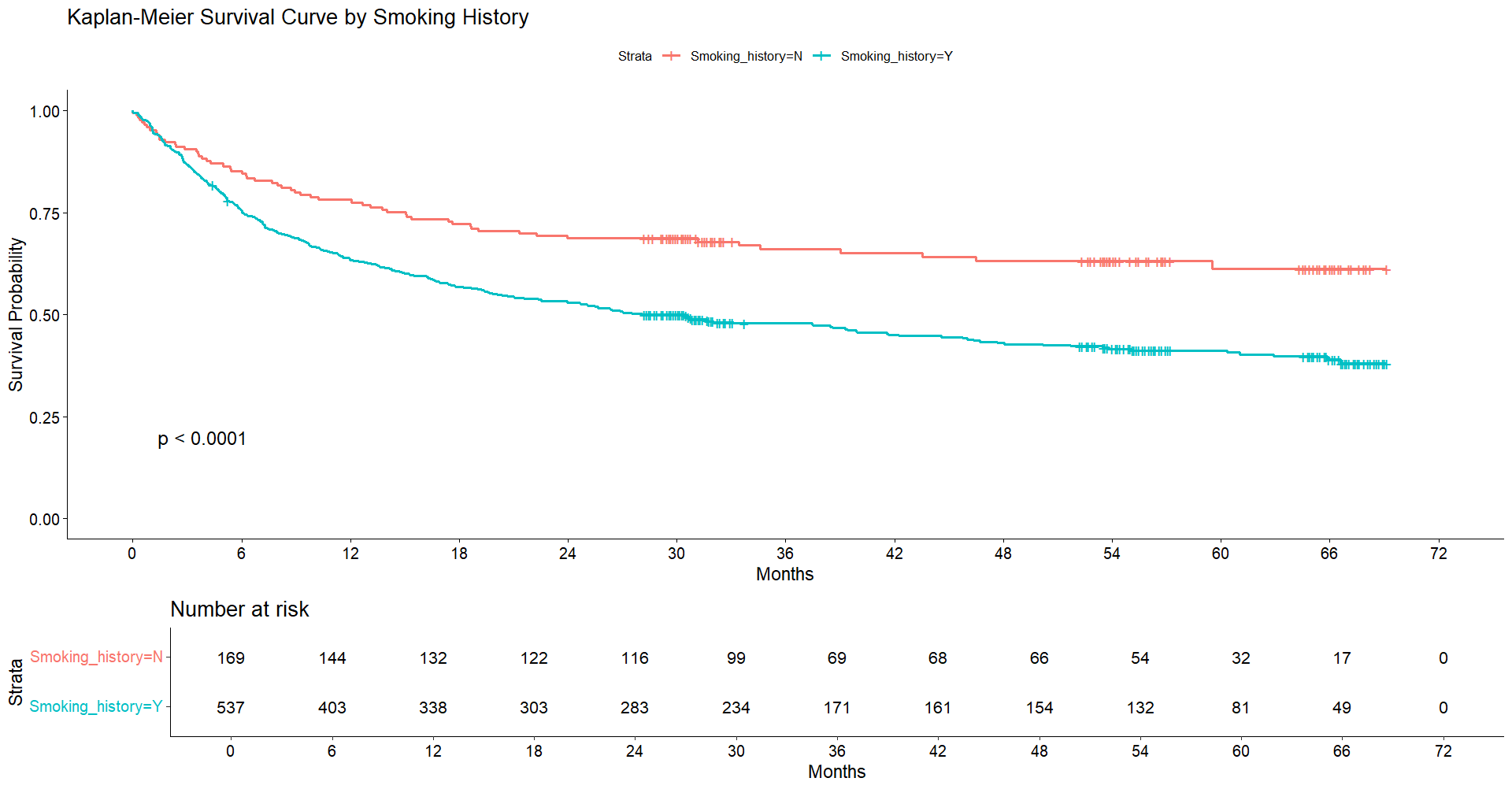


Supplementary Figure 9: Kaplan-Meier Curve by Smoking status of patients in the study (Y = Current or previous smoking, N = Never) One patient had missing smoking status information.


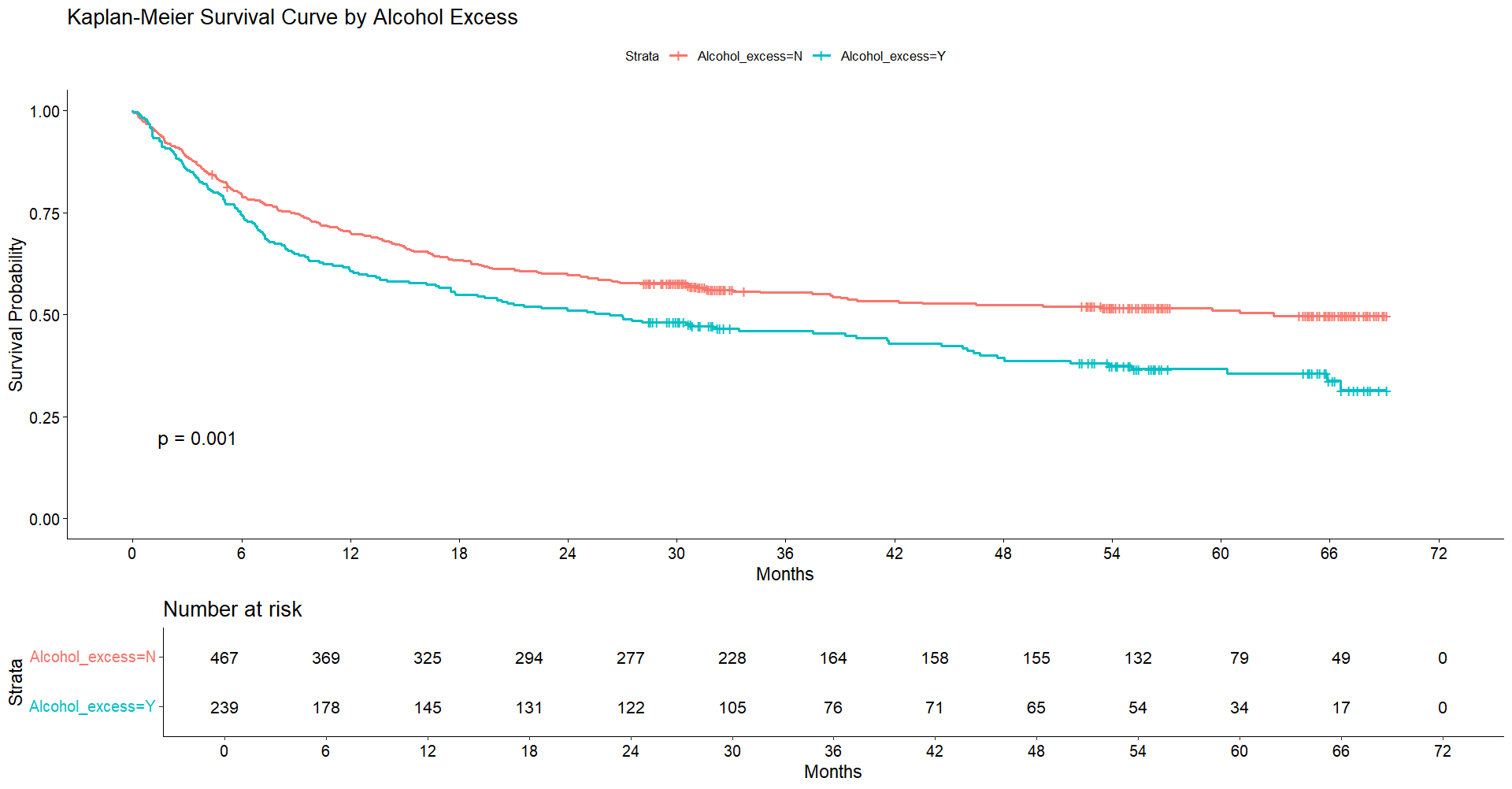


Supplementary Figure 10: Kaplan-Meier Curve by Alcohol excess (> 14 units of alcohol a week) of patients in the study (Y = Current or previous alcohol excess, N = Never) One patient had missing alcohol excess information.


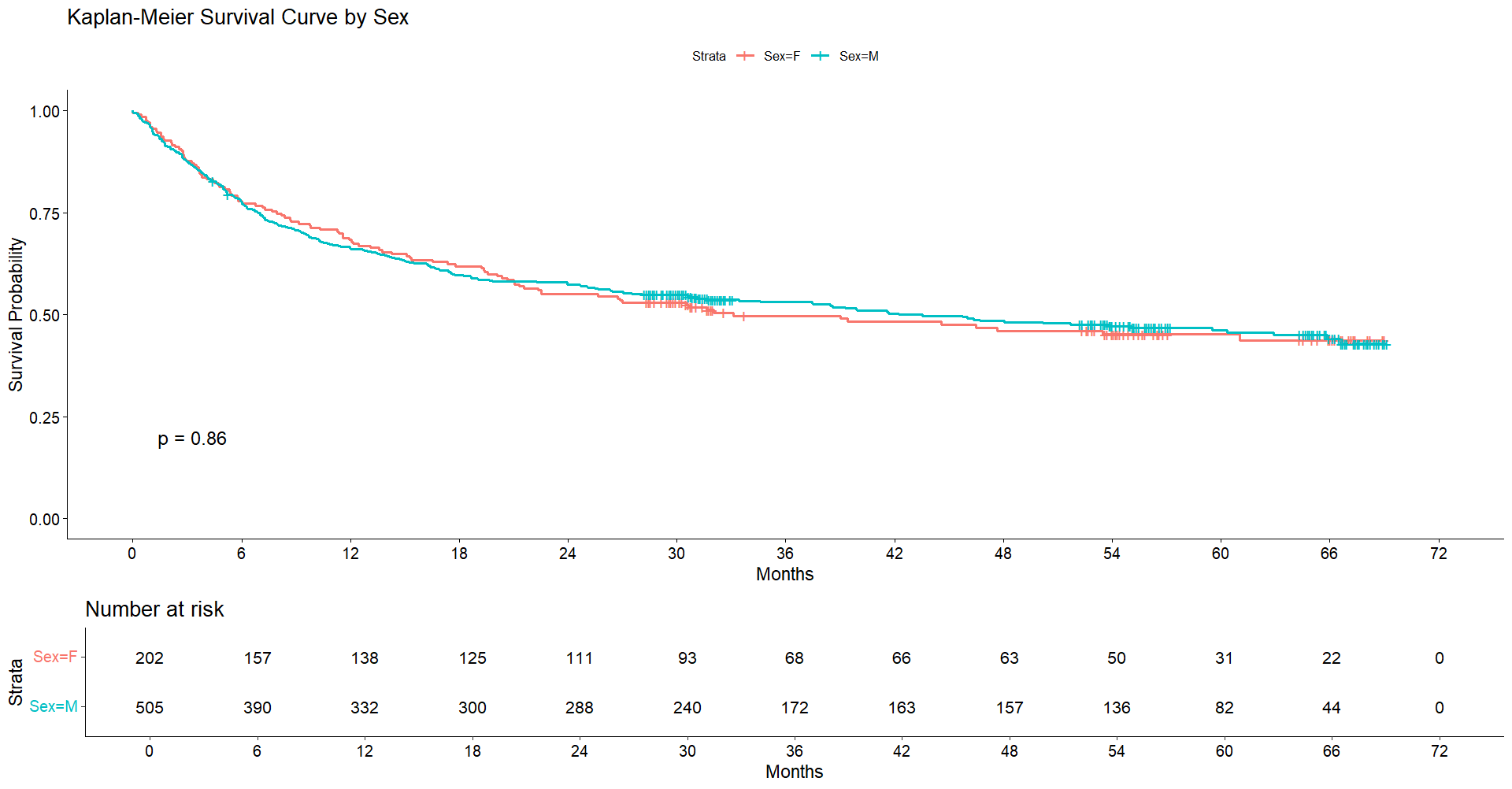


Supplementary Figure 11: Kaplan‐Meier Survival Curve stratified by Sex in the west of Scotland among the 2019, 2020 and 2022 cohorts.


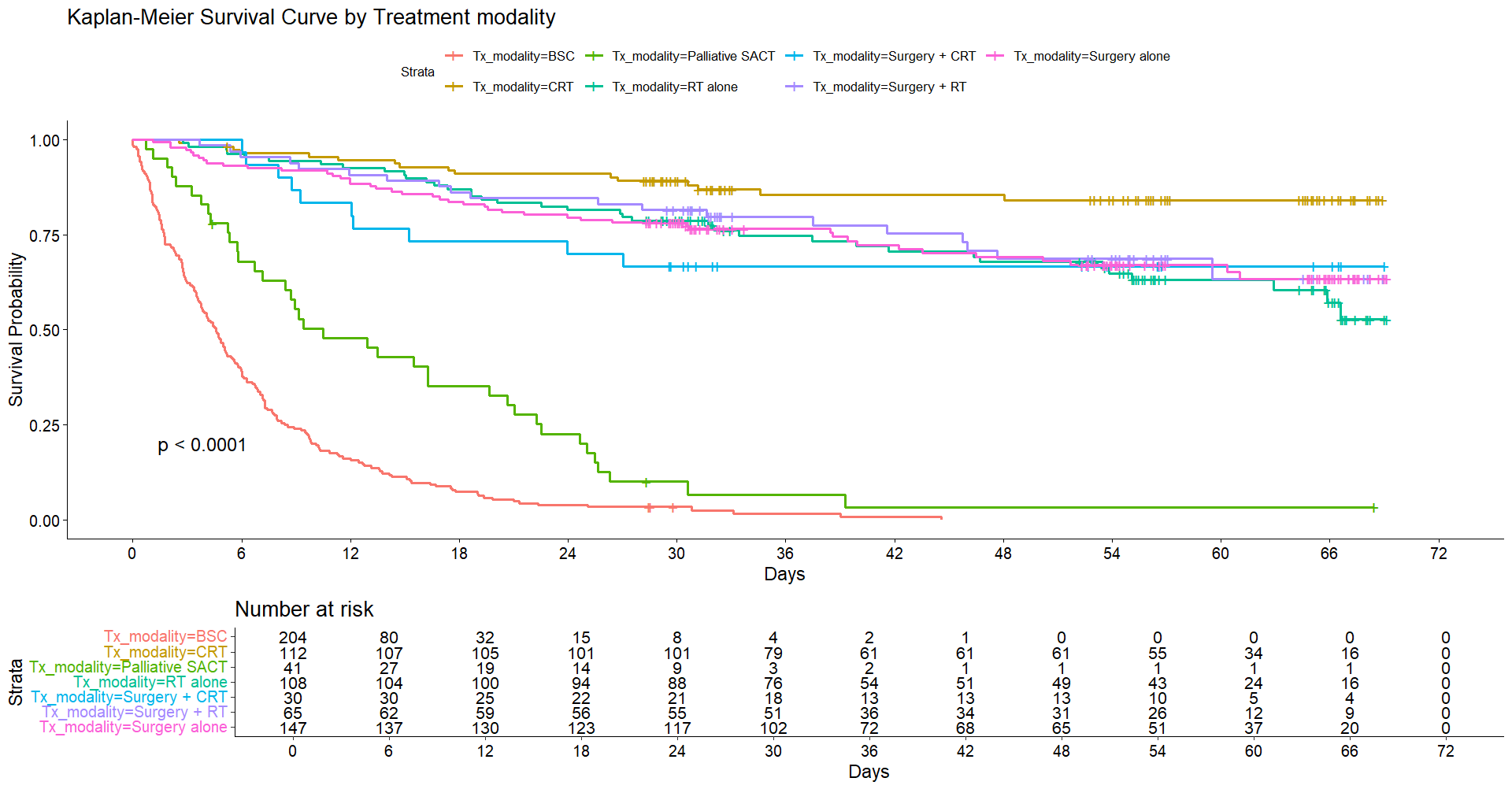


Supplementary Figure 12: Kaplan-Meier Curve by treatment delivered to patients in the study.

BSC = Best Supportive Care, SACT = Systemic Anti-cancer Therapy, CRT = Chemo-radiotherapy, RT = Radiotherapy
